# Histological Inflammation in Human Ureter either Healthy or Fitted with Double-Pigtail Stent or a Thin 0.3 F Suture Thread: A Preliminary Study

**DOI:** 10.1155/2020/1204897

**Published:** 2020-07-02

**Authors:** Benoît Vogt, Ilham Chokri

**Affiliations:** ^1^Department of Urology, Polyclinique De Blois, 1 Rue Robert Debré, 41260 La Chaussée Saint-Victor, France; ^2^Laboratory of Anatomocytopathology, 1 Av Prof Alexandre Minkowski, 37170 Chambray-Lès-Tours, France

## Abstract

**Background:**

Ureteral stent intolerance reduces patients' quality of life. It has been suggested that changes in the shape of stents could decrease discomfort. In previous studies, the innovative pigtail-suture stent (i.e., JFil® or MiniJFil®) with a thin 0.3 F suture thread significantly decreased stent-related symptoms. Fortuitously, a dilation of the ureter containing the sutures was discovered. In addition, no inflammation was seen on the ureter wall around the suture in endoscopy. In this preliminary study, we assessed ureteral inflammation in the human ureter when it was healthy or when fitted with a double-pigtail stent or a thread.

**Materials and Methods:**

After consent and inclusion of patients in the protocol, fifteen segments of ureters were collected during cystectomy procedures for bladder tumors. Ureteral inflammation was assessed on the histological section stained with hematoxylin-eosin. Histological grading (cumulative range of 0 to 6) assessing inflammation was performed on the ureter section for mucosa inflammation and inflammation in the muscle layer.

**Results:**

A marked ureteral inflammatory reaction was observed in all cases of ureters fitted with a double-pigtail stent with a mean inflammation score of 4.8 ± 0.4. The ureter fitted with the thin suture thread showed inflammation in only one case with a mean inflammation score of 1.8 ± 1.3 (*p*=0.001).

**Conclusion:**

Although the study was limited by the small number of patients, it confirmed that the double-pigtail stent induced ureteral inflammation in all cases and the thin 0.3 F suture thread caused less ureteral inflammation than the double-pigtail stent. The concept of material reduction within the urinary tract seems necessary in order to decrease mucosal irritation. The JFil® or the MiniJFil® thread could meet this requirement.

## 1. Introduction

Double-pigtail stents are frequently implanted in the ureter in urological practice, but ureteral stent intolerance reduces patients' quality of life. The symptoms may be largely due to bladder irritation caused by the stent and by the reflux during bladder voiding [1].

By decreasing the amount of material within the bladder, it should be possible to attenuate the stent-related symptoms [2–4].

To minimize the amount of material left in the bladder, a pigtail-suture stent (i.e., JFil® or MiniJFil®) was evaluated [2, 5]. With these innovative stents, the bladder loop is replaced with a thin suture thread (not more than 0.3 French). The suture thread results in the presence of only small amounts of material in the bladder. Moreover, the absence of an internal channel probably also limits renal reflux. With the pigtail-suture stent, the patients were improved [2, 5].

Fortuitously, a clear dilation of the ureter containing the sutures was discovered. We observed a ureteral diameter two to three times larger than the contralateral ureter on CT. In addition, no inflammation was seen on the ureter wall around the suture in endoscopy [2, 5]. These surprising properties led us to create the MiniJFil®, which is reduced to a thread attached to a simple loop of a pigtail stent [5, 6]. With the suture thread, the entire ureter can be dilated without inflammation, and the luminal vacuity of the ureter is preserved. Thus, the MiniJFil® may facilitate insertion of the ureteral access sheath and accelerate removal of stone fragments, and finally, MiniJFil® may improve outcome [6].

There are few studies on the interactions between the ureteral stent and the ureter, especially the human one. Several studies showed a marked ureteral inflammatory reaction when pigs were fitted with double-pigtail stents [7–9]. With the MiniJFil®, the absence of the inflammation of the ureteral mucosa in endoscopy motivated the decision to explore the ureteral histology. In this preliminary study, the objective was to determine if the thin 0.3 F suture thread caused less ureteral inflammation than the double-pigtail stent as suggested by the endoscopic appearance of previous studies [2, 5].

## 2. Materials and Methods

The study design has been approved by the French Ethical Committee (2017.09.02 bis). From September 2017 to September 2019 in a single institution, fifteen patients about to have a cystectomy with ileal conduit urinary diversion agreed to be included in the protocol and signed an informed consent form. There was no selection or exclusion, and all patients were included, even those with large tumors. Fifteen segments of ureters were gathered during cystectomy.

If gathering a human ureteral segment fitted with a double-pigtail stent is feasible, it is exceptionally rare for a ureter with a thin thread. In urological practice, cystectomy with removal of a part of the ureter is the only pathology to obtain a healthy ureteral segment fitted with a thread. The analysis of the ureters fitted with a thread can be altered by the presence of a pelvic tumor. To avoid a bias, the comparison with unstented or stented ureters was made with patients who also had a pelvic tumor.

Patients requiring no ureteral drainage served as unstented ureter controls. Patients requiring ureteral drainage for obstructive ureteral orifice were fitted with a 7 F double-pigtail stent. Patients without ureteral stents but requiring cystoscopic control before cystectomy were fitted with a MiniJFil® with the aim of facilitating the suture of the ureter in the ileal conduit urinary diversion.

The MiniJFil® stent was previously used in another study [5, 6]. In the procedure, a polyurethane double-pigtail stent (double-loop ureteral stents, 4.8 F, 26 cm, Coloplast) was sectioned perpendicularly to the main axis, just outside of the renal loop. The sectioned part is then cut parallel to the main axis to form a beveled tail that is 2 cm long. A polypropylene suture (Ethicon monofilament polypropylene suture; gauge size U.S.P.1; 0.1 to 0.15 mm; 5–0) perforates the loop and the end of the tail. The distal end of the MiniJFil® consists of two 0.3 F sutures, and each suture is approximately 36 cm long.

Ureters were fixed in 4% formalin, embedded in paraffin, serially cut, and stained with hematoxylin-eosin. As suggested by the endoscopic appearance, the ureteral mucosa looked normal in patients with a thread [2, 5]. The histological analysis was, therefore, focused on the inflammation of mucosa. Analysis of the muscular layer helped evaluate the wall of the ureter in depth. Histological grading assessing inflammation was performed on the ureter section. Following a standard evaluation protocol [9], each section was inspected for 2 characteristics: mucosa inflammation and inflammation in the muscle layer. A 4-point scale assessed the degree of change, with 0 indicating no change and 3 indicating severe changes, for each of the above characteristics (cumulative range of 0 to 6). A score was assigned by an experienced histopathologist (I.C.) blinded to the source of each specimen. The data were presented as mean ± SD and were analyzed using the Mann–Whitney test. Values of *p* < 0.05 were considered significant.

## 3. Results

Fifteen patients had bladder cancer, and three of which were sarcomas (5 women and 10 men, mean age 69.7 ± 9.6 years). Nine patients had preoperative chemotherapy, and 1 patient had surgery, chemotherapy, and radiotherapy for rectal cancer. Each group included at least a large tumor.

Five patients had no ureteral obstruction and did not require drainage with a stent. In five patients, an obstruction was located in the ureteral orifice, and these patients were fitted with an indwelling 7 F double-pigtail stent 1 to 2 months before and until cystectomy. Five patients were fitted with the MiniJFil® at least two weeks before and until cystectomy.

All ureteral segments of ureters were iliac and were gathered about 10 cm above the ureteral orifice during cystectomy. No tumor was observed on these ureteral segments.

**Figure 1:**
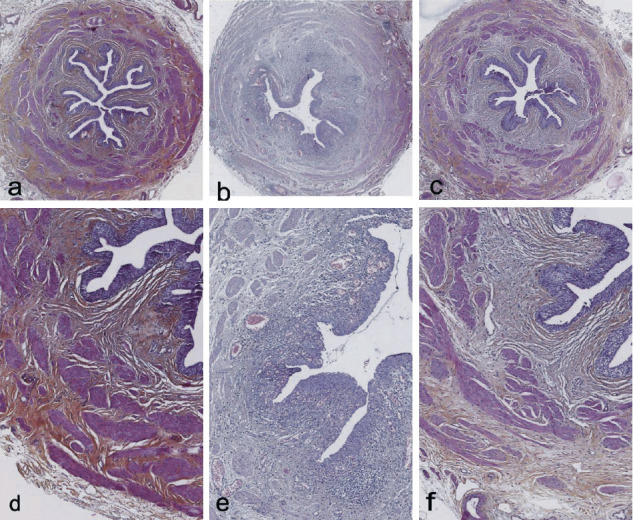
Histological appearance with hematoxylin-eosin staining. (a–c) Original magnification x3. (d–f) Original magnification x7. (a, d) Unstented control ureter showing no inflammation. (b, e) Ureter with the double-pigtail stent showing a marked ureteral inflammatory reaction. (c, f) Ureter with 0.3 F suture thread showing no inflammation.

Of the five unstented control segments, four showed no inflammation (Figures 1(a) and 1(d)). A marked ureteral inflammatory reaction was observed in all cases of ureter fitted with a double-pigtail stent (Figures 1(b) and 1(e)). In the ureter fitted with the thin 0.3 F suture thread, inflammation was observed in one case only (Figures 1(c) and 1(f)).

In cases of the ureter fitted with a double-pigtail stent, the mean inflammation score (4.8 ± 0.4) was significantly different from that of unstented control segments (1.4 ± 1.1; *p*=0.009) or that of the ureter fitted with the thin 0.3 F suture thread (1.8 ± 1.3; *p*=0.001).

In the ureter fitted with the thin 0.3 F suture thread, the mean inflammation score was not significantly different from that of unstented control segments (*p*=0.73).

## 4. Discussion

There are few studies on the interactions between the ureteral stent and the ureter, especially the human one. Janssen at al. described severe intraluminal dilation, scuffed epithelium, and pronounced submucosal edema and inflammation in all ureters when the pigs were fitted with a 6 F double-pigtail stent [8]. Olweny et al. observed important edema of the bladder mucosa and fibrosis in the lamina propria at 1 week when Yucatan minipigs were fitted with a 7 F double-pigtail stent [9]. Natalin et al. found that histological grading scores for inflammation and fibrosis were more pronounced when the ureter or the ureteral orifice of pigs was fitted with a 6 F double-pigtail stent [7].

**Figure 2:**
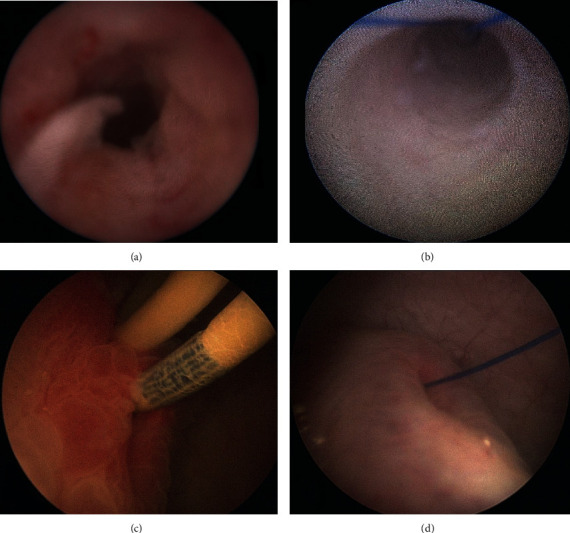
Endoscopic appearance. (a) Appearance of the inflamed ureter around the double-pigtail stent after stent removal. (b) Dilation of the ureter without inflammation after pigtail-suture stent implantation. (c) Inflamed ureteral orifice around the double-pigtail stent. (d) Dilated ureteral orifice without inflammation after pigtail-suture stent implantation.

Natalin noted the interaction between stent material and the ureter as a mechanism of inflammation and aperistalsis. With a particular shape of stents, the authors observed a smaller reduction in peristalsis, possibly reflecting its lesser interaction with ureteral tissue [7]. As suggested by endoscopic appearance of previous studies (Figure 2) [2, 5], the thin thread is expected to decrease interactions with the ureteral and bladder mucosa, and histological inflammation.

Although the study was limited by the small number of patients, the use of a semiquantitative scale made it possible to specify the alterations of the ureteral wall less subjectively. In the preliminary study, the thin 0.3 F suture thread caused less ureteral inflammation than the double-pigtail stent. With the MiniJFil®, a healthy ureter in its entirety can be dilated without inflammation and the ureteral dilation could reduce ureteral stenosis. Further studies could explore the possibility of reducing postoperative ureteroileal stenosis using MiniJFil®.

In the ureter fitted with the thin 0.3 F suture thread or in the unstented control segments, inflammation was observed in only one case. The patient with a history of rectal cancer was fitted with a MiniJFil®. Radiotherapy could explain the ureteral inflammatory reaction observed in that particular case. However, no explanation was found for the control group patient with ureteral inflammation. There was considerable heterogeneity in patients due to tumor volume, treatments, and ureteral obstructions. Lymphatic obstruction and edematous infiltration due to the tumor mass burden may explain the heterogeneity of these results. Moreover, Janssen et al. already suggested some level of cross talk between the two sides that requires further investigation [8]. However, this apparent good result contrasts with the ureteral inflammation with double-pigtail stents and confirms that thinner material is in favour of good mucosa ureteral tolerance.

The concept of material reduction within the bladder seems necessary in order to decrease bladder mucosal irritation and stent-related symptoms [2–4]. However, the concept of material reduction within the ureter should focus attention in order to improve outcome. Sfoungaristos et al. showed that ureteral stents, even if they were removed just before extracorporeal shock wave lithotripsy, decreased the stone free rate. Edema formation with decreasing functional ureteral lumen diameter and low ureteral peristalsis may minimize the likelihood of stone passage [10]. Kuebker et al. noted that the overall rate of stone passage around the 6 F double-pigtail stent was 8% and the passage rate for ≥7 mm stones was 0% [11]. In comparison with the double-pigtail stent, the luminal burden of the ureter only fitted with a pigtail-suture stent was reduced to the threads (i.e., MiniJFil®). The absence of edema around the suture and the luminal freedom may facilitate the elimination of stone fragments [6].

If the thinness of the thread is expected to limit the irritation of the ureter as suggested by the endoscopic appearance, the mechanisms or the local factors involved in the ureteral dilation are still unknown. As Janssen et al. suggested, improving our understanding of specific molecular mechanisms leading to stent-associated ureteral dysfunction may identify targets for future therapeutic agents [8].

There were several limitations to the present study. First, the small number of patients requires more targeted studies but, if gathering the ureteral segment fitted with a double-pigtail stent is feasible, it is exceptionally rare for a ureter with a thin thread because cystectomy is the only indication. Second, the heterogeneity of the bladder diseases and edematous infiltration due to the tumor mass burden may explain the heterogeneity of results.

## 5. Conclusion

Although the study was limited by the small number of patients, it confirmed that the double-pigtail stent induced ureteral inflammation in all cases and the thin 0.3 F suture thread caused less ureteral inflammation than the double-pigtail stent.

The concept of material reduction within the urinary tract seems necessary in order to decrease mucosal irritation. The search for the ideal ureteral stent could focus on the shortening and the thinning of the intraureteral and bladder material at least in the healthy segments of the ureter. The JFil® or the MiniJFil® thread could meet this requirement.

## Data Availability

The data used to support the findings of the study are available from the corresponding author upon reasonable request.

## References

[1] Chew B. H., Lange D. (2016). Advances in ureteral stent development. *Current Opinion in Urology*.

[2] Vogt B., Desgrippes A., Desfemmes F.-N. (2015). Changing the double-pigtail stent by a new suture stent to improve patient’s quality of life: a prospective study. *World Journal of Urology*.

[3] Nestler S., Witte B., Schilchegger L., Jones J. (2020). Size does matter: ureteral stents with a smaller diameter show advantages regarding urinary symptoms, pain levels and general health. *World Journal of Urology*.

[4] Vogt B. (2020). A new customized ureteral stent with nonrefluxing silicone end-piece to alleviate stent-related symptoms in malignant diseases. *Urology*.

[5] Vogt B., Desgrippes A., Desfemmes F.-N. (2014). Sondes JFil et MiniJFil: progrès décisifs dans la tolérance des sondes urétérales et propriétés inattendues du fil urétéral. *Progrès en Urologie*.

[6] Vogt B., Desfemmes F. N., Desgrippes A. (2016). MiniJFil®: a new safe and effective stent for well-tolerated repeated extracorporeal shockwave lithotripsy or ureteroscopy for medium-to-large kidney stones?. *Nephro-Urology Monthly*.

[7] Natalin R. A., Hruby G. W., Okhunov Z. (2009). Pilot study evaluating ureteric physiological changes with a novel “ribbon stent” design using electromyographic and giant magnetoresistive sensors. *BJU International*.

[8] Janssen C., Buttyan R., Seow C. Y. (2017). A role for the hedgehog effector Gli1 in mediating stent-induced ureteral smooth muscle dysfunction and aperistalsis. *Urology*.

[9] Olweny E. O., Portis A. J., Sundaram C. P. (2000). Evaluation of a chronic indwelling prototype mesh ureteral stent in a porcine model. *Urology*.

[10] Sfoungaristos S., Gofrit O. N., Pode D. (2015). History of ureteral stenting negatively affects the outcomes of extracorporeal shockwave lithotripsy. results of a matched-pair analysis. *Prague Medical Report*.

[11] Kuebker J. M., Robles J., Kramer J. J., Miller N. L., Herrell S. D., Hsi R. S. (2019). Predictors of spontaneous ureteral stone passage in the presence of an indwelling ureteral stent. *Urolithiasis*.

